# Increase in hnRNPA1 Expression Suffices to Kill Motor Neurons in Transgenic Rats

**DOI:** 10.3390/ijms242216214

**Published:** 2023-11-11

**Authors:** Bo Huang, Xionghao Liu, Tingting Zhang, Qinxue Wu, Cao Huang, Xu-Gang Xia, Hongxia Zhou

**Affiliations:** 1 Department of Pathology, Thomas Jefferson University, 1020 Locust Street, Philadelphia, PA 19107, USA; 2 Department of Environmental Health Sciences, Robert Stempel College of Public Health & Social Work, The Center for Translational Sciences, Florida International University, Port St. Lucie, FL 34987, USA

**Keywords:** hnRNPA1, motor neuron disease, ALS, rats, transgenic rodents

## Abstract

A dominant mutation in hnRNPA1 causes amyotrophic lateral sclerosis (ALS), but it is not known whether this mutation leads to motor neuron death through increased or decreased function. To elucidate the relationship between pathogenic hnRNPA1 mutation and its native function, we created novel transgenic rats that overexpressed wildtype rat hnRNPA1 exclusively in motor neurons. This targeted expression of wildtype hnRNPA1 caused severe motor neuron loss and subsequent denervation muscle atrophy in transgenic rats that recapitulated the characteristics of ALS. These findings demonstrate that the augmentation of hnRNPA1 expression suffices to trigger motor neuron degeneration and the manifestation of ALS-like phenotypes. It is reasonable to infer that an amplification of an as-yet undetermined hnRNPA1 function plays a pivotal role in the pathogenesis of familial ALS caused by pathogenic hnRNPA1 mutation.

## 1. Introduction

Amyotrophic lateral sclerosis (ALS) is a common neurodegenerative disease characterized by progressive degeneration of motor neurons and subsequent denervation atrophy of skeletal muscles [1,2]. While most cases of ALS arise sporadically without a clear cause, familial ALS results from specific mutation in ALS-associated genes [3,4,5,6,7,8,9,10,11,12]. In several families affected by ALS, the mutation in the *hnRNPA1* gene is genetically linked to the disease [12], implicating it as a causal factor in familial ALS. HnRNPA1 plays a crucial role in regulating RNA processing and interacts with various proteins to perform diverse functions [13,14,15,16]; however, the precise mechanisms through which hnRNPA1 mutation leads to disease remain to be determined.

HnRNPA1, alongside the ALS-related genes TDP-43 and FUS [6,7,8,9,10,11], belongs to the ribonucleoprotein family. These three ribonucleoproteins share common disease characteristics, including an autosomal inheritance pattern and the development of prominent proteinopathies. Interestingly, both the normal and mutant forms of TDP-43 and FUS produce similar disease phenotypes when overexpressed in transgenic rodents [17,18,19,20,21,22,23,24]. This observation suggests that the exacerbation of yet-unidentified functions of TDP-43 and FUS is involved in the pathogenesis of ALS. In our quest to elucidate the mechanistic underpinnings of hnRNAP1-caused ALS, there exists an imperative need to delineate the precise relationship between alterations in hnRNPA1 function and the pathogenesis of familial ALS.

There lacks an understanding of how the exclusive presence of pathogenic hnRNPA1 within motor neurons impacts neuronal function in intricate systems like transgenic rodents. We previously showed that selective expression of pathogenic TDP-43 solely in motor neurons is adequate to initiate autonomous neuron death [25]. However, it is worth noting that neurons may undergo unique degenerative processes when exposed to mutations in different genes [26,27]. Hence, it is of significant interest to investigate whether the mere presence of excess hnRNPA1 within motor neurons can elicit neuronal dysfunction and lead to the development of an ALS-like phenotype in an animal model.

To determine whether increased expression of wildtype hnRNPA1 has a detrimental or beneficial impact on neurons, we generated transgenic rats that specifically overexpressed wildtype hnRNPA1 within motor neurons. The targeted overexpression of wildtype hnRNPA1 in motor neurons resulted in the severe loss of motor neurons and subsequent atrophy of skeletal muscles due to denervation, recapitulating the key characteristics of ALS. These findings suggest that an excess of hnRNPA1 expression leads to neurotoxicity rather than neuroprotection and that restricted overexpression of hnRNPA1 in motor neurons is sufficient to induce autonomous neuron death in transgenic rats.

## 2. Results and Discussion

**Figure 1:**
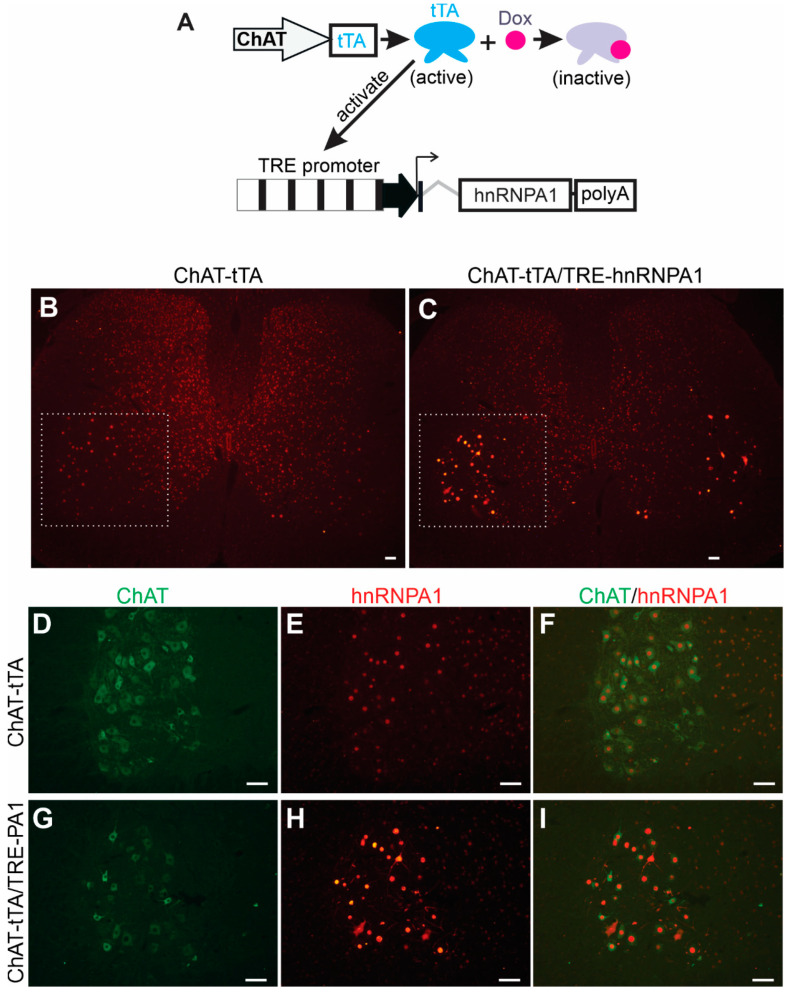
Targeted overexpression of hnRNPA1 in the motor neurons of transgenic rats. (**A**) A diagram depicted a method to selectively increase hnRNPA1 expression in the spinal motor neurons of transgenic rats. Rat hnRNPA1 cDNA was inserted downstream of the TRE promoter. TRE-hnRNPA1 transgenic line was bred with the ChAT-tTA transgenic line established in the lab to produce the double transgenic rats that expressed hnRNPA1 exclusively in spinal motor neurons upon removal of Doxycycline (Dox) from their drinking water. (**B**–**I**) Immunofluorescence staining confirmed increased hnRNPA1 expression in motor neurons in the ventral horns of these double transgenic rats (**C**,**G**–**I**). The ventral horns of lumbar spinal cords (**B**,**C**) were magnified to show the colocalization of hnRNPA1 (stained with an hnRNPA1 specific antibody) with the motor neuron marker ChAT (**D**–**I**). Both ChAT-tTA single (**B**,**D**–**F**) and ChAT-tTA/TRE-hnRNPA1 double (**C**,**G**–**I**) transgenic rats underwent Dox removal at 35 days of age and were examined for transgene expression at 60 days of age. Scale bars: 50 µm (**B**,**C**) and 25 µm (**D**–**I**).

To determine the effects of elevated hnRNPA1 expression on neuronal functions in a systematic manner, we used a well-established tetracycline-inducible transgene expression system within our laboratory [25]. This inducible system allows us to precisely control the timing of transgene expression, ensuring that even a highly toxic transgene can be successfully passed on from transgenic founders to subsequent generations, as previously demonstrated [25,28]. The system involves two independently generated transgenic rat lines (Figure 1A): one line expresses tetracycline-controlled transcriptional activator (tTA) from a selected promoter, and the other line harbors the target transgene under the control of the tetracycline-responsive element promoter (TRE) [29]. The spatial and temporal patterns of target transgene expression are governed by the activity of the promoter regulating the tTA transgene. In other words, the location and timing of TRE promoter activation are determined by the activity of the promoter driving the tTA transgene when the tTA inducer, tetracycline, or its analog Doxycycline (Dox), is not present. To achieve precise expression of the target transgene exclusively within lower motor neurons, we established a transgenic rat line, known as ChAT-tTA, in which the tTA transgene is controlled by the motor neuron-specific ChAT promoter, as previously described [25]. For the targeted expression of the hnRNPA1 transgene in motor neurons, we developed a new transgenic line that contained rat hnRNAP1 cDNA under the control of the TRE promoter (Figure 1A). Given the identical amino acid sequence between human and rat hnRNPA1 proteins, we selected rat hnRNPA1 cDNA for constructing the TRE-driven hnRNPA1 transgene (Figure 1A). The stable and inheritable TRE-hnRNPA1 transgenic rat line was then bred with the established ChAT-tTA rat line to generate double transgenic offspring. These offspring expressed hnRNPA1 transgene specifically in spinal motor neurons after Dox was removed from the rat’s drinking water (Figure 1). Double-labeling immunofluorescence staining revealed a specific increase in hnRNAP1 immunostaining within motor neurons that were located in the ventral horns of the spinal cord in double transgenic rats, as compared to single transgenic rats (Figure 1B–I). The data indicate that wildtype hnRNPA1 was substantially and selectively overexpressed within the motor neurons of ChAT-tTA and TRE-hnRNPA1 double transgenic rats.

**Figure 2:**
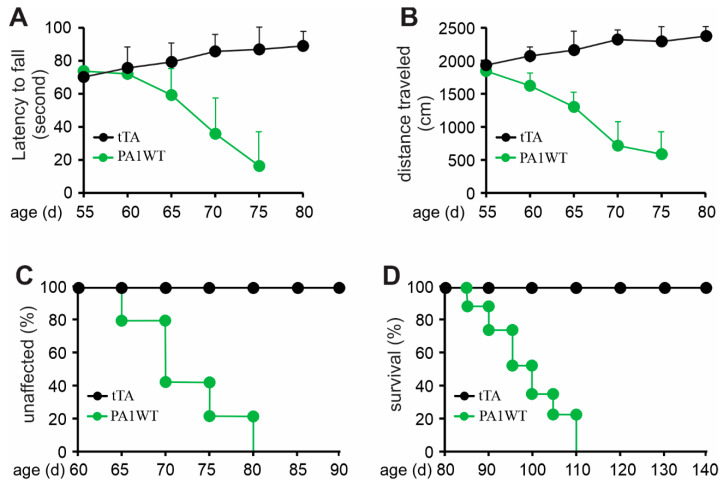
Overexpression of wildtype hnRNPA1 selectively in motor neurons caused progressive paralysis in transgenic rats. (**A**) The Rotarod test indicates a gradual decrease in psychomotor activity in the ChAT-tTA/TRE-hnRNPA1 double transgenic rats expressing wildtype hnRNPA1 (PA1WT) compared to ChAT-tTA single transgenic rats (tTA). (**B**) The Open field assay demonstrates a progressive decline in mobility in the double transgenic rats (PA1WT) compared to the single transgenic rats (tTA). (**C**,**D**) Diagrams depicted the rates of disease onset and animal survival for tTA single and PA1WT double transgenic rats. ChAT-tTA single (tTA) and ChAT/TRE-hnRNPA1 double (PA1WT) transgenic rats were deprived of Dox at the age of 35 days and were examined for mobility from the age of 50 days onward (n = 10).

**Figure 3:**
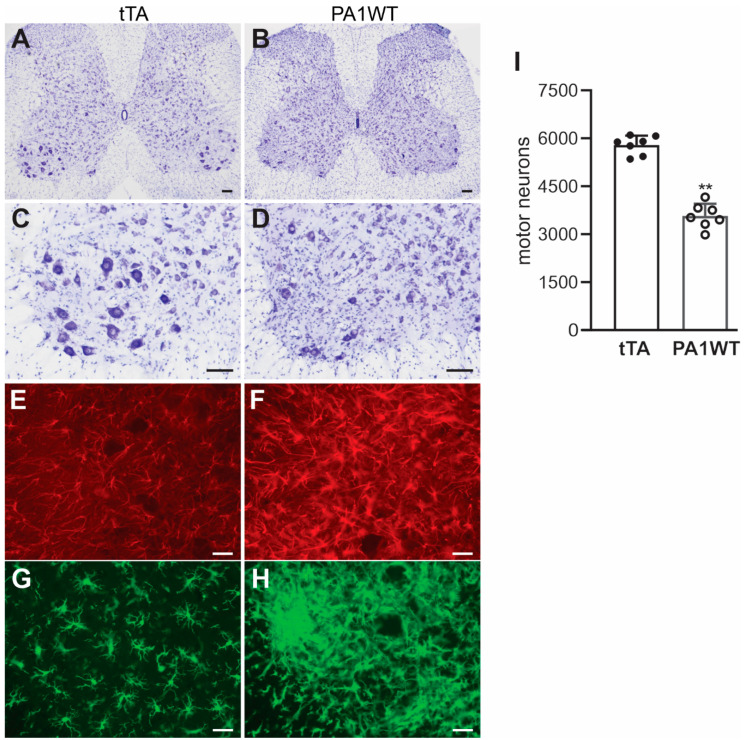
Targeted overexpression of hnRNPA1 in motor neurons caused severe motor neuron death and glial reaction in transgenic rats. (**A**–**D**) Nissl staining indicated severe motor neuron loss in the lumbar spinal cords of ChAT-tTA/TRE-hnRNPA1 double transgenic rats (PA1WT) compared to ChAT-tTA single transgenic rats (tTA). (**E**–**G**) Immunofluorescence staining revealed the presence of astrocytes (**E**,**F**) and microglia (**G**,**H**) in the ventral horns of the rat’s spinal cords. Double transgenic rats (PA1WT) were assessed during paralysis stages, while single transgenic rats (tTA) were examined at corresponding ages. Scale bars: 100 µm (**A**,**B**) and 40 µm (**C**–**H**). (**I**) Stereological cell counting quantified motor neurons (diameter > 25 µm) in the L3–L5 lumbar cords of ChAT-tTA single (tTA) and ChAT-tTA/TRE-hnRNPA1 double (PA1WT) transgenic rats, with data presented as means ± SD (n = 7). The symbol ** indicates statistical significance at *p* < 0.05.

**Figure 4:**
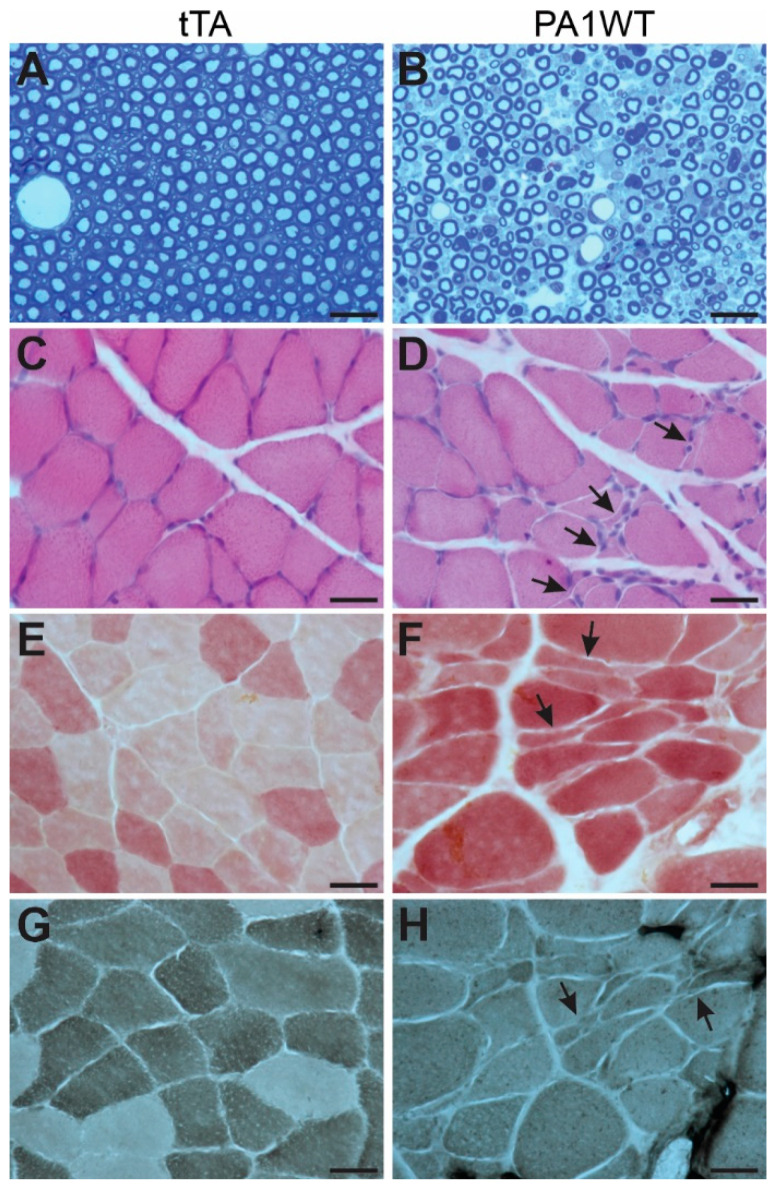
Targeted overexpression of wildtype hnRNPA1 in motor neurons caused motor axon degeneration and denervation muscle atrophy. (**A**,**B**) Toluidine blue staining revealed the structures of ventral nerve roots in ChAT-tTA single (tTA) and ChAT-tTA/TRE-hnRNPA1 double (PA1WT) transgenic rats. Motor axons in L3 ventral roots underwent severe degeneration in the double transgenic rats (**B**) compared to the single transgenic rats (**A**). (**C**–**H**) Denervation atrophy of gastrocnemius muscles was revealed by H&E staining (**C**,**D**), histochemistry for nonspecific esterase (**E**,**F**), and ATPase staining (**G**,**H**). Arrows point to atrophied muscle fibers. Double transgenic rats (PA1WT) were assessed during paralysis stages, while single transgenic rats (tTA) were examined at corresponding ages. All scale bars: 30 µm.

Selective overexpression of wildtype hnRNPA1 specifically within motor neurons resulted in a gradual decline in motor function, as evidenced by the Rotarod test and Open Field activity assay (Figure 2A,B). In the case of double transgenic rats expressing hnRNPA1, they experienced a rapid onset of paralysis by 70 days of age, with a subsequent demise around 95 days of age (Figure 2). Pathological examination revealed a severe loss of motor neurons in the ventral spinal cord horns of double transgenic rats during the paralysis stages (Figure 3). This motor neuron loss was confirmed through unbiased stereological cell counting Figure 3I), and, concurrently, glial cells, including astrocytes and microglia, exhibited pronounced activation in response to motor neuron death (Figure 3E–H). Furthermore, motor axons in the ventral roots exhibited significant degeneration, as indicated by toluidine blue staining (Figure 4A,B). Consequently, due to the death of motor neurons, skeletal muscles underwent denervation atrophy, which was detectable through H&E staining, ATPase activity assays, and histochemistry for nonspecific esterase (Figure 4C–H). The ChAT-tTA transgenic rats did not possess the hnRNPA1 transgene but did have the tTA transgene, making them a suitable negative control for hnRNPA1 overexpression. Furthermore, the TRE-hnRNPA1 single transgenic rats did not exhibit any observable phenotype or pathological alteration. Consequently, it is clear that the motor neuron pathology and ALS phenotype arose from the deliberate overexpression of hnRNPA1 within motor neurons, and not from insertional mutations caused by the transgene. These findings demonstrate that the overexpression of wildtype hnRNPA1 exclusively within motor neurons is sufficient to trigger cell-autonomous motor neuron death and ALS-like symptoms in the rat model.

## 3. Materials and Methods

### 3.1. Creation and Breeding of Transgenic Rats

Animal studies were approved by the Institutional Animal Care and Use Committees at Thomas Jefferson University and at Florida International University. Animal research was conducted in accordance with NIH guidelines.

Transgenic rats with a Sprague-Dawley genomic background were generated and maintained as previously described [21,22,23,25]. The ChAT-tTA transgenic line has been previously characterized [25]. For creating TRE-hnRNPA1 transgenic rats, the open reading frame of rat *hnRNPA1* was inserted between the tetracycline-responsive element (TRE promoter) and SV40 poly (A) signaling sequence [20]. PCR was used with specific primers (forward primer: 5′-TTGTTTGTGGATCGCTGTGA; reverse primer: 5′-GACAAACTTCACGTCAGGGT) to identify TRE-hnRNPA1 transgenic rats. Quantitative PCR was employed to determine the number of transgene copies, following the protocol established in the lab [28]. TRE-hnRNPA1 transgenic rats were bred with ChAT-tTA transgenic rats to produce offspring with both transgenes. Breeding rats were given Doxycycline (Dox) in their drinking water to suppress hnRNPA1 transgene expression during the embryonic and postnatal development of offspring, as previously described [28]. Weaned rats continued to receive Dox-containing water (50 µg/mL) until they reached 35 days old, at which time point the hnRNPA1 transgene was allowed to be activated upon Dox withdrawal.

### 3.2. Rat Behavior Analyses

As determined for the ChAT-tTA transgenic line used here [25], TRE-hnRNPA1 transgene became fully active 10 days after the discontinuation of Dox treatment. Rats at 50 days of age underwent behavioral assessments, including the Open Field assay and Rotarod test. For the Open Field assay, individual rats were placed in a 27.9 cm × 29.9 cm chamber equipped with infrared beams to track their location and locomotor activity. Data were recorded in 10 min trials, and the average travel distance within each 10 min interval was calculated. In the Rotarod test, rats were assessed for motor coordination and agility using an Economex accelerating Rotarod (Columbus Instruments, Columbus, USA). Initially, rats were trained to remain on the rotating rod at a constant speed of 10 rpm. After a week of training, they were tested on the rod with a starting speed of 10 rpm, which gradually increased by 0.4 rpm/second. The time each rat remained on the rotating rod was measured, and the average latency to fall from three trials was used for behavioral analysis.

Disease onset was defined as an irreversible reduction in travel distance during the Open Field assay or in latency to fall on the Rotarod test. Disease end-stages were characterized by the inability to retract two or more legs or the inability to right itself when placed on its side. Paralysis was defined as the dragging of legs or the inability to retract individual legs.

### 3.3. Cresyl Violet Staining and Stereological Cell Counting

Neurons in the spinal cord of transgenic rats were visualized using Cresyl violet staining and were quantified through stereological cell counting, following established methods [28]. The lumbar spinal cord (specifically, segments L3–L5) was dissected and sectioned into 30 µm thick cross-sections. Motor neurons with a diameter exceeding 25 µm were counted in every 10th section on both sides of the spinal cord, amounting to 15–20 sections per rat.

### 3.4. Histochemistry, Immunofluorescence Staining and Toluidine Staining

Established procedures were followed to assess denervation atrophy in gastrocnemius muscle, as previously described [23,25]. This involved histochemical staining using ATPase and nonspecific esterase techniques, as well as hematoxylin and eosin staining. For analysis of the lumbar spinal cord from the transgenic rats, 20 µm thick cross-sections were prepared. These sections were subjected to immunostaining using specific primary antibodies, including a mouse monoclonal antibody against GFAP (Agilent Technologies, Santa Clara, CA, USA), a rabbit antibody against Iba-1 (Wako Chemicals, Richmond, VA, USA), a goat anti-ChAT antibody (Millipore, Temecula, CA, USA), and a rabbit polyclonal antibody against hnRNPA1 (Proteintech, Rosemont, IL, USA). Immunofluorescence staining was employed, and the resulting images were visualized and documented using a Nikon fluorescence microscope.

Toluidine blue staining was used to depict the structure of motor axons in the ventral roots of spinal nerves, following well-established laboratory procedures [28].

For non-statistical analyses, such as histochemistry and immunostaining, these procedures were routinely conducted on three individual animals to verify the data’s reproducibility.

### 3.5. Statistical Analysis

An unpaired *t*-test was employed to identify statistically significant differences in spinal motor neurons between ChAT-tTA/TRE-hnRNPA1 double transgenic rats and ChAT-tTA single transgenic rats. A *p*-value of less than 0.05 was considered indicative of statistical significance.

## 4. Conclusions

Increased expression of the normal hnRNPA1 gene specifically within motor neurons results in a gradual decline in motor neurons and the subsequent atrophy of skeletal muscles, recapitulating the pathological characteristics of ALS. These discoveries indicate that an enhanced, yet unidentified, function of hnRNPA1 plays a pivotal role in the pathogenesis of ALS caused by pathogenic hnRNPA1 mutation.

## Data Availability

All data supporting the conclusion of this study are included within the article.
